# To Disclose or Not to Disclose: A Multi-stakeholder Focus Group Study on Mental Health Issues in the Work Environment

**DOI:** 10.1007/s10926-019-09848-z

**Published:** 2019-08-13

**Authors:** E. P. M. Brouwers, M. C. W. Joosen, C. van Zelst, J. Van Weeghel

**Affiliations:** 1 Department Tranzo, Tilburg School of Social and Behavioral Sciences, NETHLAB, Tilburg University, P.O. Box 90153, 5000 LE Tilburg, The Netherlands; 2 Department Human Resource Studies, Tilburg School of Social and Behavioral Sciences, NETHLAB, Tilburg University, Tilburg, The Netherlands; 3 Department of Psychiatry and Neuropsychology, Maastricht University, Maastricht, The Netherlands; 4 Phrenos Center of Expertise, Utrecht, The Netherlands; 5 Parnassia Group, The Hague, The Netherlands

**Keywords:** Discrimination, Stigma, Employment, Mental illness, Disclosure

## Abstract

*Purpose* Whether or not to disclose mental illness or mental health issues in the work environment is a highly sensitive dilemma. It can facilitate keeping or finding paid employment, but can also lead to losing employment or to not being hired, because of discrimination and stigma. Research questions were: (1) what do stakeholders see as advantages and disadvantages of disclosing mental illness or mental health issues in the work environment?; (2) what factors are of influence on a positive outcome of disclosure? *Methods* A focus group study was conducted with five different stakeholder groups: people with mental illness, Human Resources professionals, employers, work reintegration professionals, and mental health advocates. Sessions were audio-taped and transcribed verbatim. Thematic content analysis was performed by two researchers using AtlasTi-7.5. Results were visually represented in a diagram to form a theoretical model. *Results* Concerning (dis-)advantages of disclosure, six themes emerged as advantages (*improved relationships, authenticity, work environment support, friendly culture)* and two as disadvantages *(discrimination and stigma).* Of influence on the disclosure outcome were: Aspects of the *disclosure process, workplace factors, financial factors, and employee factors*. Stakeholders generally agreed, although distinct differences were also found and discussed in the paper. *Conclusion* As shown from the theoretical model, the (non-)disclosure process is complex, and the outcome is influenced by many factors, most of which cannot be influenced by the individual with mental illness. However, the theme ‘Aspects of the disclosure process’, including subthemes: *who to disclose to, timing, preparation, message content* and *communication style* is promising for improving work participation of people with mental illness or mental health issues, because disclosers can positively influence these aspects themselves.

## Introduction

Despite the fact that employment is beneficial for health [1], participation of people with mental illness in the workforce is problematic [2, 3]. Here, the role of social stigma and discrimination as a barrier to employment is increasingly being acknowledged [4]. Whereas one study showed that sometimes workers are being treated more positively because of their mental illness [5], others have found that people with mental health disorders frequently report discrimination in the work setting [6, 7], and that employers have been found to often hold negative attitudes towards people with mental illness [8, 9] and engage in discriminatory behaviour [10]. Fear of being stigmatized in the workplace, is a major reason for non-disclosure of mental health issues to a supervisor [11, 12]. However, non-disclosure precludes supervisor support and workplace adjustments [13], which both are important for *staying* at work.

Whether or not disclosure of mental illness in the work environment yields positive outcomes for the employee or job applicant depends on many factors, including the views and behaviour of other stakeholders. The aim of the present study was to explore views of five different stakeholder groups. Specifically, the research questions were: (1) what do stakeholders see as advantages and disadvantages of disclosing a mental illness or mental health issues in the work environment?; (2) what factors are of influence on a positive outcome of disclosure (in favour of the discloser)?

## Methods

In December 2016 and January 2017 five (homogeneous) focus groups were conducted, including 27 participants in total. Groups consisted of: (1) People with mental illness; (2) Human Resources (HR) managers, involved in hiring decisions; (3) employers; (4) work reintegration professionals; and (5) mental health advocates. The focus groups were part of a larger project in which the CORAL decision aid [14] was translated and adapted for use in The Netherlands.

### Participants

To select stakeholders involved in the employment of people with mental illness, purposive sampling was used. Participants were recruited with help from mental health care staff, an occupational health service, a network of employers, a peer support organisation for people with mental illness, other researchers, and the Dutch Anti-Stigma Association. Seven people with mental illness, 15 employers, eight work reintegration professionals, and two HR managers declined participation due to a lack of time or interest. The recruitment procedure resulted in the following focus groups:

I. A focus group of six *people with mental illness*. All were male, previously diagnosed with one or more of the following diagnoses: autism spectrum disorder, schizophrenia, ADHD, addiction problems and psychosis. Four had paid employment, two were unemployed.
II. A focus group of five *employers,* four of whom were male. They represented companies in consultancy, catering, cleaning, construction and the plastics industry. One represented a small company (< 50 employees), all others a large company (> 250 employees). The average number of years they worked as a manager was 5, ranging from 3 to 12.
III. A focus group of four *Human Resources (HR) managers.* All were female. They worked for moderate (> 50 employees) to large (> 250) organisations, in education, one in a local municipality and two in (different) health care organisations. The number of years they had worked as HR manager dealing with hiring decisions was 1, 6, 17 and 25, respectively.
IV. A focus group of four *mental health advocates (hereafter: advocates),* who had mental illness and who worked as volunteers, pro-actively fighting stigma for the Dutch Anti-Stigma Association. Three of them were females. Previous diagnoses included dysthemia, schizoaffective disorder, posttraumatic stress disorder, recurrent depressive disorder, personality disorder not otherwise specified, and attention deficit disorder. Three had paid employment.
V. A focus group of eight *work reintegration professionals (hereafter: professionals)*, including five job coaches, two occupational physicians and one occupational social worker. Six of them were female. The average number of years they had worked as a work reintegration professional was 13, ranging from 6 to 32.

In each focus group, participants received a gift voucher of 10 euros (about 8, 75 GPB) for their participation.

### Focus Groups

The 2-h focus groups took place in a conference room at Tilburg University (The Netherlands) except for the one of mental health advocates, which took place at the Dutch Anti-Stigma association in Amersfoort. All focus groups were conducted by two researchers (CvZ, EB, JW, DV). Each group was facilitated by an experienced researcher (EB or CvZ), who guided the group discussion, and an observer (CvZ, JvW, DV). Focus groups started with the general question what stakeholders’ views were on whether or not it is wise for an individual with mental illness or mental health issues to disclose them in the work environment. Topics discussed referred both to disclosure of psychiatric disorders, and to common mental health issues, such as high stress and burnout syndrome. The topics discussed can be found Table 1.

**Table 1:** Topic list used in the focus groups

1. What are stakeholders’ views on whether or not it is wise to disclose mental illness, or mental health issues, in a work environment?
2. What do stakeholders perceive as *advantages* of (non-)disclosure for the individual with mental illness or mental health issues?
3. What do they perceive as *disadvantages* of (non-)disclosure for the individual with mental illness or mental health issues?
4. What aspects play a role in a positive or negative disclosure outcome for people with mental illness or mental health issues?
5. *How* can they best disclose?
6. What is the role of the individual versus the work environment?
7. What do stakeholders find important *from their own perspective*, regarding disclosure of mental illness or mental health issues in the work environment?

### Coding, Data Analysis and Interpretation of the Data

All discussions were audiotaped and transcribed verbatim to enable deductive and inductive thematic content analysis [15]. Transcripts were anonymised before analyses were performed. To increase reliability, each transcript was repeatedly read and coded by two researchers independently, (EB, MJ, JvW) using the software package ATLAS-ti, version 7.5.16. The research questions were used as framework (pre-defined categories) i.e. (1) what participants viewed as advantages and disadvantages, and (2) what they viewed as factors of influence on a (for the employee) positive outcome. Subsequently, themes and subthemes within these predefined categories were created by the method of constant comparison in which different codes were compared and the relationship between codes was explored to detect emerging themes [15]. This process was executed by the researcher (EB) who clustered the codes and defined emergent themes. Next, these results were discussed by three researchers (MJ, JvW, EB) until consensus was derived on interpretation of the themes. Separate code lists and emergent themes were created per focus group to be able to identify differences and similarities between the groups. In case comparable themes emerged in the focus groups, the same theme titles were used to enhance visibility of similarities and differences. Results were visually represented in a diagram [16], to form a theoretical model.

### Ethical Considerations

All participants were informed verbally and in writing about the study before signing a written informed consent form. They were aware that participation was voluntary, that their information would be kept confidential and would be used for research. Prior to the study, a statement of the Medical Ethics Committee (*Medische Ethische Toetingscommissie Brabant*) was obtained declaring that the project did not fall under the Dutch *Medical Research Involving Human Subjects Act (WMO)*, for which reason a more elaborate ethical review was not necessary.

## Results

### Research question 1: What do Stakeholders See as Advantages and Disadvantages of Disclosing a Mental Illness in the Work Environment?

Four themes were identified as advantages, and two as disadvantages. These were: (1) disclosure can improve relationships at work; (2) authenticity is important for wellbeing at work; (3) the work environment can help; (4) disclosure can help create a friendly and inclusive workplace culture; (5) disclosure can lead to discrimination; and (6) disclosure can lead to stigma.

### Theme 1 (Advantage): Disclosure can Improve Relationships at Work

All groups agreed that disclosure improves understanding of the disclosers’ behavior and situation, and as such can improve relationships at work. Moreover, it was believed that honesty would be appreciated by others in the work environment, and would yield respect.

Employer, (male):

“I would advise an employee with mental illness or mental health issues to disclose [to the manager], and to indicate what you have already done about it yourself, and what you need as an employee to function well. I believe that once you’re open about it, that 9 out of 10 times it will be really appreciated by the employer”.

### Theme 2 (Advantage): Being Allowed to be Who You are (Authenticity) is Important for Wellbeing at Work

This theme was strongly supported and discussed by three groups: people with mental illness, advocates and professionals. Concealment of mental illness or mental health issues made people feel dishonest, guilty, and exhausted. In contrast, being able to be yourself in the workplace felt like a weight was taken of their shoulders, and was also seen as important for optimal work performance.

Advocate (female):

“That I did not have to lie anymore, that was great. That I did not have to tell them I had the flu again, whenever we had a team building activity. I had started to feel so bad about that…”

Person with mental illness (male):

“I believe that if you cannot be your true self…. whether it concerns mental illness or something else, … you will never be able to reach your peak performance. And you will never feel great, which I believe is a prerequisite for doing your job well….

The stress that people experienced from having to conceal their mental illness at work could be so high that it in itself increased the risk of developing mental health issues.

Person with mental illness (male):

“In my case, it [concealment] led to an extra crisis. A new crisis started just because I simply could not tell.”

Whereas for the people with mental illness and the advocates, being allowed to be yourself was regarded as extremely important for wellbeing at work, this theme was not discussed by employers or HR managers.

### Theme 3 (Advantage): The Work Environment can Help

All groups strongly endorsed this theme as an important advantage of disclosure, especially the role the work environment can play in the prevention of adverse outcomes, e.g. by providing work adjustments and understanding.

Employer (male):

“I believe it is important to disclose. I, as an employer, would appreciate it, so I can be of help”.

### Theme 4 (Advantage): Disclosure can Help Create a Friendly and Inclusive Workplace Culture

The effect of disclosure on enhancing a friendly and inclusive workplace culture was neither discussed by employers nor by HR managers. However, it was mentioned as an important advantage of disclosure in the remaining three focus groups, especially the effect on reducing stigma on the work environment.

Advocate (female):

“[once you disclose] you get great talks. It opens a dialogue and you notice that there is more mutual trust. Because you share information that makes you vulnerable, you get some in return. Then you have a very different interaction than if you are both hiding information”.

### Theme 5 (Disadvantage): Disclosure Can Lead to Discrimination

In all groups, the risk of discrimination was discussed as a major disadvantage of disclosure. This generally referred to the discloser being seen as a financial risk for the employer. The HR professionals came up with considerable more ways in which disclosure could lead to discrimination than the other groups. These were all related to reducing the employers’ financial risk and included the following: trying to get rid of the employee, no continuation of a temporary contract after it finishes, not hiring, offering the discloser only temporary contracts, a shorter contract or lower salary. HR managers were the only group discussing this theme (discrimination) after disclosure as a useful phenomenon. They viewed it as a successful hiring decision, for which they carried responsibility in their own jobs. From their *own* point of view, they were in favor of disclosure during the hiring period, because this would enable them to avoid hiring the discloser and protect their employer from financial risk.

HR manager (female):

“..I notice that I wear two hats… because if you ask me “What do you want, as an employer?” I want to know everything. Yes, seriously! Because that allows me to assess the risks. But if I wear my hat as an employee, I say: if you know that it does not influence your work, it is best not to say anything because then you will not be treated differently”.

### Theme 6 (Disadvantage): Disclosure can Lead to Stigma

In all groups, the risk of being stigmatized was discussed as a major disadvantage of disclosure. Generally, the groups agreed that disclosure can create social distance or yield disrespect from others at work. Similar to the theme of discrimination, most examples of stigma were mentioned by the HR managers. These included: (a) increased chances of gossip, or unpleasant verbal reactions by others (e.g. jokes); (b) lower performance expectations of the employee (subsequently yielding low efforts of work related support; (c) too much focus on what goes wrong at work and attribution of mistakes to the mental illness; (e) the discloser has to perform better than others in order to be seen and treated as equal.

### Research Question 2: Which Factors are of Influence on a Positive Outcome of Disclosure?

Here, four main themes emerged from the data. These were: (1) aspects of the disclosure process; (2) workplace factors; (3) financial factors; (4) employee factors.

### Theme 1: Aspects of the Disclosure Process

When discussing factors of influence on a favorable outcome, the disclosure process itself was one of the most extensively discussed factors, and 5 important subthemes emerged within that theme.

The first was *who to disclose to*. Generally it was agreed that disclosure should primarily be directed towards the supervisor (unless there is a bad relationship). Moreover, overall it was agreed that selective disclosure rather than being completely open to everyone at work would yield better outcomes.

Professional (job coach, male):

“[Prior to a job interview] it is important to discuss with the client to what extent he is going to disclose…because every company has different [management] layers, and not everyone has to know everything. But someone.., in a higher [management] position, who has the power to make important decisions? Yes I would inform him/her, together with my client of course.”

The second subtheme was *the timing of disclosure.* Generally, it was believed that if work functioning is not or minimally affected by the symptoms, for the employee’s wellbeing and employability it is best not to disclose, in order to avoid stigma and discrimination. However, all groups agreed that disclosure is necessary when health problems start to influence work performance and preferably even before, if preventive measures can be taken. Furthermore, four groups strongly believed that disclosing a mental illness or mental health issues during the hiring period (e.g. job interview) substantially decreases the risk of being hired.

Mental health advocate (female):

“An HR manager wants to avoid [financial] risk of course. I am a risk, that is actually what I have been called, a risk for the company”.

Employer (male):

“If you apply for a job… by saying something like that [i.e. disclosing]… I think that is a ‘no go’. Then they [employers] will say: ‘thanks for warning us’”.

HR manager (female):

“During the hiring period, it is a rat run. It is the survival of the fittest….and having a [mental health] condition means reduced employability and possible [financial] risk”.

All groups believed it was best to first build up a relationship and earn respect, before disclosing.

Employer (male):

“I don’t think you should choose a moment [for disclosure], you should earn it.”

In contrast to the other groups, the work reintegration professionals believed disclosure was much better than concealment:

Professional (job coach, female):

“If you know that work adjustments would help this employee…, well than it is great if these can be discussed, because this will just increase the chances of success”

However, professionals also had a personal interest in their clients’ disclosure, as it made their jobs easier.

Professional (job coach, male):

“[If the employee does not want to disclose]… it makes it very difficult for me to help… Then you automatically get to the point where you try to promote disclosure”.

In addition, disclosure was regarded as beneficial for their own professional relationships with their clients’ employers:

Professional (job coach, female):

“I once had a kleptomaniac as a client, and her disorder was not entirely under control yet. I found it so difficult because an employer was very willing [to offer her a job] but she did not want me to tell him…… I felt so bad towards the employer, because I could not tell him… Then something was stolen and he called me up and said ‘We think she did it’, and I could still not say anything… I felt so miserable about it”.

The third subtheme was *preparation of the disclosure process*. Several groups discussed that disclosure would yield better results if well thought out and prepared. It was suggested that others (e.g. a coach) could help by increasing self-esteem and focusing on qualities, and that the form of disclosure (e.g. one-on-one, or a presentation for a group) should suit the discloser’s personality and communication style.

The fourth subtheme was *the content of the disclosure message*. Almost all groups believed the discloser should be specific about under what conditions he/she would be best able to perform at work, considering the health problems. Also, they believed that the disclosure message should preferably be like a sales pitch: strongly emphasizing one’s qualities. The stakeholders also believed that only information with direct relevance for adequate work performance should be disclosed, and that the discloser should be reticent about additional medical or personal information. Solely mentioning the diagnosis can instantly lead to labelling and stigma, as was shown from this quote from the HR managers:

HR manager (female:)

“The moment I hear a [mental health] condition during a job interview, it is stored in my memory… After that, the job applicant can talk all he wants, but I have already heard it”.

The final subtheme regarding the disclosure process was *communication style*. This theme referred to showing a respectful tone and attitude towards the work environment, and emerged in the employers’, HR managers and work reintegration professionals groups. Here, the ‘give and take’ aspect of relationships was emphasized, which was an important and central theme in the employers’ group. It was believed that disclosure message should show that the employee is also considerate of the employers’ needs instead of having a claiming, self-centered attitude.

HR manager (female):

“We once had an employee who had been with us for 40 years. When she was 60 years old she was diagnosed with ADHD. Well, from that moment on, she leaned backwards and said she couldn’t do this because she had ADHD. I thought: “You have had this for 40 years and now you need to lean backwards?” ….I believe it should be from both sides, I don’t like the attitude of ‘I want to be open, that is who I am, so I just throw it at you’”.

Whereas all other groups believed that many aspects of the disclosure process itself (e.g. who, how, message content) were of crucial effect on its outcome, the mental health advocates did not discuss any of these aspects, except for timing.

### Theme 2: Workplace Factors

The second major theme that was believed to be of influence on a (for the discloser) favorable outcome was *workplace factors*. Generally, the groups believed that *the level of responsibility of the job* would be a determining factor. More negative outcomes of disclosure were expected in high responsibility jobs (e.g. chief executive officer) than in low responsibility jobs (e.g. cleaner). Furthermore, the *social climate* was also believed to be of influence on disclosure outcomes. Some organizations have an inclusive workplace philosophy, which was believed to be beneficial for disclosure. Finally, it was mentioned that in some *sectors*, where more ‘macho’ workplace cultures exist (e.g. construction) disclosure may yield less positive outcomes.

### Theme 3: Financial Factors

Overall, it was believed that in a poor economy, chances to be hired strongly diminish after disclosure. Also the probability of losing work was believed to increase after disclosure. In contrast, it was believed that if employers receive financial incentives (e.g. from the government) they will be more open to hiring people with mental illness or mental health issues.

### Theme 4: Employee Factors

The final theme that was believed to be of influence on disclosure outcomes concerned *employee factors*, which consisted of three important subthemes. First, it was generally believed that the type of mental health issues or diagnosis matters. For instance, it was expected that autism, ADHD, burnout and PTSS would yield more positive reactions after disclosure than schizophrenia, bipolar disorder or substance use disorder. The second subtheme was *the extent to which the discloser was (still) experiencing symptoms*. It was believed that if the employee has recovered and seems in control, the work environment would react more positively to the disclosure. The final employee related subtheme was the degree of self-esteem and empowerment. Here, two groups stressed that higher self-esteem, empowerment and negotiation skills will yield better outcomes after disclosure.

**Fig. 1:**
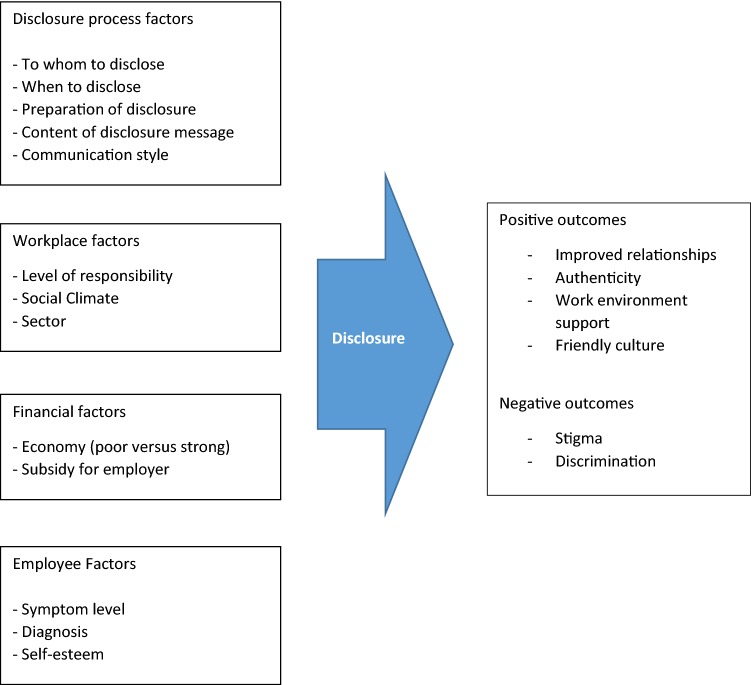
Influencing factors and outcomes of the disclosure process

All major themes found for research questions 1 and 2 are graphically depicted in Fig. 1.

## Discussion

The aim of the study was to explore different stakeholder perspectives on advantages and disadvantages of (non-) disclosure, and on factors determining a successful outcome. Generally, it was believed that disclosure can have important benefits, but if work functioning is not or minimally affected by the symptoms, for the employee’s wellbeing and employability it is best not to disclose—especially during the hiring period- in order to avoid stigma and discrimination. Whereas stakeholders generally agreed in their views, some distinct differences were also found, especially when it considered how they viewed disclosure from their *own* perspective. HR managers may form an important target group for destigmatizing interventions. From the many factors discussed that are of influence on the disclosure outcome, especially the theme ‘*Aspects of the disclosure process’* seems promising, as it entails subthemes that disclosers can influence themselves. From the results, particularly three important findings warrant further study as they may help increase work participation of people with mental illness or mental health issues.

First, by contrasting perspectives, a discrepancy emerged between HR managers on one respect, and people with mental illness and mental health advocates on the other. Specifically, all were in favour of disclosure, but for opposite reasons. HR managers were in favour of disclosure, because it enabled them to discriminate and avoid financial risk. They had mixed feelings about it, but viewed it as a core responsibility of their jobs. However, the other two groups emphasized that authenticity in the workplace was extremely important for wellbeing and work performance. The finding that concealment was associated with feeling dishonest, resulting in a weight on one’s shoulders causing stress and exhaustion has also been found in previous studies [4, 13–18]. Individuals can experience their health problem as a part of their identity, for which reason they want to disclose [19]. Whereas several studies have shown workplace authenticity to be positively related to wellbeing, job performance, job satisfaction and work engagement [17, 18], this theme was not addressed during the focus groups of employers or HR managers. This suggest that these groups do not fully realize that inclusive workplaces where workers can be authentic can be beneficial to them as well. Increasing their knowledge could enhance work participation of workers with mental illness or mental health issues. Others have also suggested employers’ knowledge regarding employees with such health issues needs to be increased [20–22], and may reduce mental health stigma in the workplace [21, 23]. Considering that HR managers came up with markedly more ways in which disclosure can result in discrimination and stigma, and that they spoke about discrimination as a useful phenomenon suggests they form an important target group for destigmatizing intervention studies aiming to reduce discrimination. However, on the other side of the discrepancy, mental health advocates were so strongly in favour of disclosure and advocacy, that they may underestimate the risk of unemployment. Indeed, they barely discussed the importance of the process of disclosure, a factor strongly acknowledged by the other groups. Other studies have also shown that planning disclosure strategically is an important way to decrease the harmful effects of stigma [23, 24]. Increasing knowledge among people with mental illness or mental health issues may therefore better prepare them to cope with stigma in the work environment and enhance their sustainable employment.

A second important finding warranting future study was that the work reintegration professionals were convinced the hiring period was a good time for disclosure, whereas all others strongly believed this was the worst time for disclosure. The finding that employment specialists are in favour of and encourage their clients’ disclosure has also been found by others [24, 25]. In line with what respondents generally believed, several studies have shown that disclosure during the hiring period resulted in fewer invitations for job interviews [10], and that greater reluctance to disclose mental illness increased chances of reemployment after 6 months, [26]. There are several explanations for this deviant opinion of work reintegration professionals. First, they had personal gain from disclosure. Disclosure made it easier for them to do their own jobs (e.g. help arrange work accommodations), and nondisclosure could harm their own valued professional relationship with employers. Second, as job coaches tended to work with long-lasting relationships with inclusive employers, they may have developed a biased view of what average employers’ attitudes are towards people with mental illness. A final explanation is that especially the job coaches worked with clients with more severe health problems. If symptoms are more severe and visible, disclosure may yield better outcomes than concealment.

Third, the findings suggest that by thorough preparation, workplace disclosers can themselves have a positive influence on the outcome. The findings are in line with those of other studies, suggesting that strategic disclosure can decrease discrimination and stigma [20, 21, 24]. This is important, as also a variety of factors were found that *cannot* be influenced by the discloser (e.g. the economy). The results provided a variety of suggestions for a successful disclosure process, regarding as who to disclose to, when, how to prepare, message content and communication style of the message. Moreover, empowerment, self-esteem and negotiation skills which were believed to be of influence on the outcome, also are aspects that can be influenced by appropriate training and preparation. Future intervention studies investigate these aspects in more detail.

Whereas this paper focused on mental health stigma, the problems discussed are not illness specific and plausibly can just as well be applied to other groups. For instance, several very recent studies have described that people with concealable physical illness, e.g. with HIV [27], diabetes [28], or a history of cancer [29] also struggle with the stigma related dilemma of whether to disclose or conceal their health problems in the work environment. Theoretical models on mental health stigma in the workplace may therefore also be helpful to improve work participation of people with other stigmatized conditions.

## Strengths and Limitations

There is limited qualitative research with employers [5] and a strength was that the present study not only included employers but also HR managers as an additional workplace stakeholder group. Moreover, many studies in this area use students as a proxy for employers, and vignettes rather than real hiring situations [5], so a strength of the study is that it concerned real stakeholders with personal and professional experience. Limitations were that some groups were relatively small, and that it can be expected that primarily respondents with an interest in the study topic participated, possibly resulting in respondents with more positive attitudes to social inclusion than average. Indeed, in the employer and work reintegration professional group, some participants disclosed a history mental problems during the focus group. However, this did not happen in the HR managers group. Moreover, insight rather than generalizability is the aim of qualitative research. An additional limitation was that despite explicit efforts, no respondents with more common mental disorders were willing to participate in the focus group. They are prevalent in the workplace, are more likely to conceal their mental health problems, and better support for them is needed [30]. Additional limitations were that the people with mental illness group and the HR group only entailed participants of one gender, and that no public sector employers were included. More differentiation within the focus groups would have been preferable and should be aimed for in further studies.

In conclusion, whereas stakeholders generally agreed in their views, some distinct differences were also found, especially when it considered how they viewed disclosure outcomes from their *own* perspective. HR managers seem an important stakeholder group whose predominantly negative attitudes and behaviour should be confirmed in future studies. As HR managers act on behalf of their organization, their attitudes and behavior may not change unless destigmatizing interventions are also aimed at the level of their organization (e.g. including management). Finally, as shown from the Fig. 1, the (non-)disclosure process is complex, and the outcome is influenced by many factors, most of which cannot be influenced by the individual. However, this study also found factors that *can* be influenced by the individual with mental illness or mental health issues and the several specific suggestions for successful disclosure warrant further investigation.
